# The Expression Patterns of p53 and p16 and an Analysis of a Possible Role of HPV in Primary Adenocarcinoma of the Urinary Bladder

**DOI:** 10.1371/journal.pone.0095724

**Published:** 2014-04-21

**Authors:** Riley E. Alexander, Sean R. Williamson, Justin Richey, Antonio Lopez-Beltran, Rodolfo Montironi, Darrell D. Davidson, Muhammad T. Idrees, Carol L. Jones, Shaobo Zhang, Lisha Wang, Qiu Rao, Jose A. Pedrosa, Hristos Z. Kaimakliotis, M. Francesca Monn, Michael O. Koch, Liang Cheng

**Affiliations:** 1 Department of Pathology and Laboratory Medicine, Indiana University School of Medicine, Indianapolis, Indiana, United States of America; 2 Department of Urology, Indiana University School of Medicine, Indianapolis, Indiana, United States of America; 3 Department of Pathology, Henry Ford Hospital, Detroit, Michigan, United States of America; 4 Department of Pathology, Cordoba University, Cordoba, Spain; 5 Institute of Pathological Anatomy and Histopathology, School of Medicine, Polytechnic University of the Marche Region (Ancona), United Hospitals, Ancona, Italy; 6 Department of Pathology, Fudan University Shanghai Cancer Center, Shanghai, China; 7 Department of Pathology, Nanjing Jinling Hospital, Nanjing University School of Medicine, Nanjing, China; Biomedical Research Foundation, Academy of Athens, Greece

## Abstract

**Background:**

Primary adenocarcinoma of the urinary bladder is rare. The molecular and cellular events leading to its pathogenesis are not well delineated. The goal of this study was to investigate p53 and p16 expression, as well as HPV status, in a relatively large series of primary bladder adenocarcinomas.

**Materials and Methods:**

Thirty six cases of urinary bladder adenocarcinoma were chosen from participating institutions. The diagnosis and available clinical history were reviewed in each case. Immunostains for p53, p16 and HPV and high-risk and low-risk HPV-ISH were performed on all tumors.

**Results:**

Patients had an average age of 61 years with a male predominance (1.5∶1 male∶female ratio). The average tumor size in cystectomy specimens was 4.3 cm. Of the cases managed by transurethral resection, 40% were pT2 at the time of diagnosis. In cystectomy specimens, 77% were either pT3 or pT4. Strong nuclear p16 expression was seen in 67% of all cases and p53 expression was present in 58% of the cases. Expression of both markers was seen in 33% of cases. Expression of p16 or p53 alone was present in 12 (33%) and 9 (25%) cases, respectively. Neither marker was expressed in only 3 (8%) of the tumors. No significant correlation between clinical variables and any of the markers we studied was identified. No HPV infection was detected in any case.

**Conclusions:**

Expression of p53 and/or p16 is very common in urinary bladder adenocarcinoma. These findings implicate a high likelihood that alterations in these cell cycle proteins contribute to the pathogenesis of these tumors. Despite frequent immunohistochemical labeling for p16, no evidence of HPV infection was found.

## Introduction

Primary adenocarcinoma of the urinary bladder is rare. It accounts for only 0.5–2% of all urinary bladder malignancies [1]–[3]. Bladder adenocarcinoma histology is most common where bilharziasis is endemic. Therefore, studies on non-bilharziasis related adenocarcinoma are even more rare than studies allowing both endemic and sporadic adenocarcinoma of the urinary bladder [4], [5]. Although adenocarcinoma usually presents at a higher stage than conventional urothelial carcinoma (72% versus 52% muscle invasive cases, respectively), survival differences are not clearly significant [6]. Histologically and immunohistochemically, primary adenocarcinomas of the urinary bladder are similar to the more common colonic adenocarcinomas [7]. This further limits the number of tumors available for bladder-specific analysis, as substantial clinicopathologic data is necessary to exclude secondary involvement by direct extension or metastasis of colorectal origin. Due to this rarity and the additional challenges faced with diagnosis, little work has been performed to investigate the molecular and cellular mechanisms responsible for tumorigenesis in this entity.

The tumor suppressor proteins p53 and p16 have been well studied and are altered in a number of human malignancies [8]–[11]. These markers have been specifically studied in urothelial carcinoma and here, too, they are altered in a significant proportion of tumors. In addition, urothelial tumors with alterations in p53 have been shown to portend a less favorable prognosis, and they are associated with decreased survival [12], [13]. A single study investigating the expression of cell cycle markers in primary bladder adenocarcinoma showed a high frequency of p53 alteration in these tumors. However, p16 was not evaluated and the patient population had a high prevalence of bilharziasis (67%) [14].

In this study, we evaluated the expression of p53 and p16 in a relatively large cohort of non-schistosome related primary bladder adenocarcinomas. Investigation of the expression pattern of these tumor suppressor proteins may shed light on the pathogenesis of this unusual malignancy. In some organ systems, such as in the uterine cervix and upper aerodigestive tract, expression of p16 is valuable as a surrogate marker for human papillomavirus (HPV) infection [11], [15]–[19]. To address this hypothesis, we also performed HPV in situ hybridization (ISH) and HPV immunohistochemistry on all tumors.

## Methods

### Patients and Ethical Statements

Thirty-six cases were selected from the surgical pathology case files of the participating institutions. Hematoxylin and eosin (H&E) stained slides were reviewed from each case to confirm the diagnosis and to select adequate tumor for immunohistochemical staining and ISH. Clinical and pathologic records were reviewed for each case, with particular attention to exclude any case with a known adenocarcinoma of another organ. This precaution was taken to eliminate the possibility of investigating metastatic or extrinsic disease. The Institutional Review Boards of the participating institutions (Indiana University, Cordoba University, and Polytechnic University of the March Regions) approved this study. All tissues were collected for diagnostic purposes and were anonymized for the use in the current study. Therefore, no informed consents were obtained

### Immunohistochemistry and HPV in situ Hybridization

All immunohistochemical studies were performed on 5 µm thick sections of formalin-fixed paraffin embedded tissue. Immunostaining for p53 was performed using a monoclonal anti-p53 antibody (D0-7, prediluted; Dako, Carpinteria, CA). An anti-p16 mouse monoclonal antibody using the CINtec Histology kit (antibody E6H4, prediluted; CINtec Histology, Westborough, MA, USA) was used for detection of p16 expression. Only nuclear immunoreactivity was deemed positive for either antibody. Extent of staining was calculated on a percentage basis and then stratified into a 4-tier scoring system: 0 (no staining); 1+ (1–33% staining); 2+ (34–66% staining); and 3+ (>66% staining).

HPV in situ hybridization was performed on all cases using Inform HPV II Family 6 Probe (detecting HPV genotypes 6 and 11) and HPV III Family 16 Probe (detecting HPV genotypes 16, 18, 31, 33, 35, 39, 45, 51, 52, 56, 58, and 66) (Ventana Medical Systems, Inc, Tucson, AZ). Immunohistochemical studies for HPV used an anti-HPV mouse monoclonal antibody (Clone K1H8, 1∶50; Dako, Carpinteria, CA). The HPV antibody can detect HPV subtypes 6, 11, 16, 18, 31, 33, 42, 51, 52, 56, and 58. Nuclear staining was considered positive for HPV. Appropriate negative and positive controls were used for each immunostain and ISH slide.

## Results

Patients in this study with available demographic information had an average age of 61 years (range of 32–87 years). There was a moderate male predominance (1.5∶1 male to female ratio). The average tumor size was 4.3 cm. The majority of cases (61%) presented as pathological tumor stage (pT) 1 or 2 disease. Of these pT1 or pT2 cases, 68% of them were found in either biopsy material or transurethral resection specimens, where pT2 is the limit of pathological assessment. When limiting the analysis to resection specimens, 66% of cases were found to be either pT3 or pT4. Full results of tumor staging in this series are found in **Table 1**. In all but two cases, the tumor was well-to-moderately differentiated. One of the poorly differentiated tumors had predominately signet-ring cell morphology. Three cases showed mucinous/colloid morphology.

**Table 1:** Tumor Classification by Stage (n = 36).

Stage	pT1	pT2	pT3	pT4
All tumors	12	10	11	3
Only biopsy or transurethral resection specimens*	10	5	Not applicable	Not applicable
Only resection specimens	2	5	11	3

*Diagnosis of stage pT3 or pT4 disease is not possible on these types of specimens

**Figure 1:**
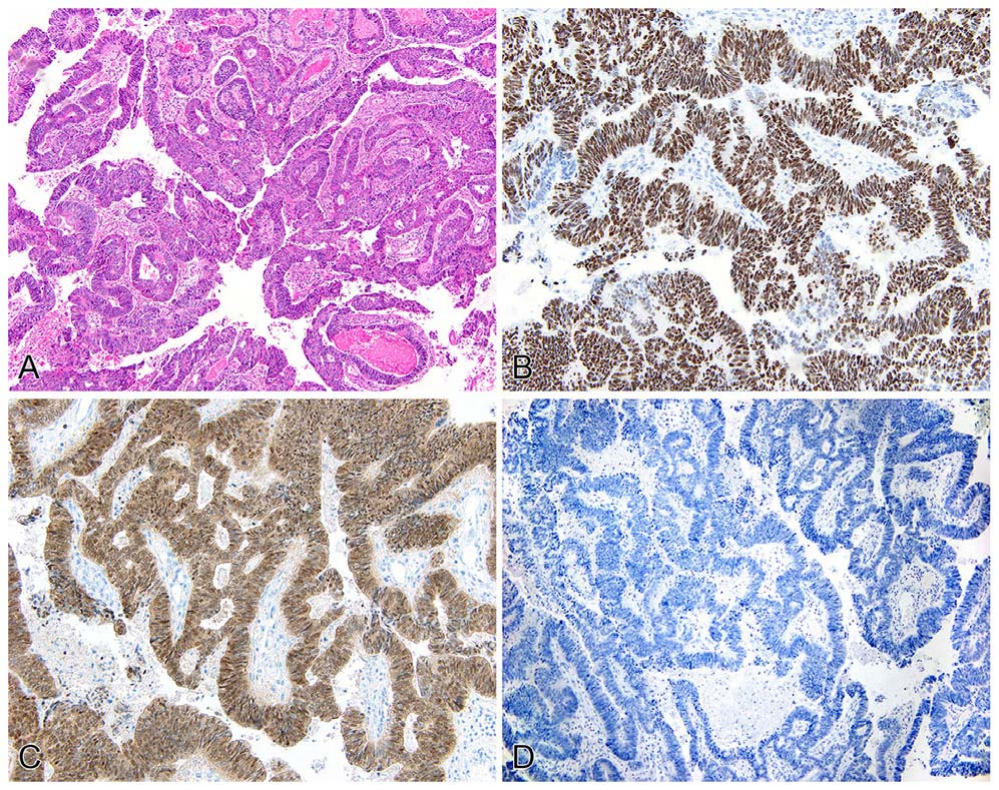
Primary adenocarcinoma of the urinary bladder (pT2). H&E displaying intestinal-type architecture (A). p53 staining shows diffuse, strong nuclear reactivity (B). p16 shows both strong nuclear and cytoplasmic staining in tumor cells (C). Immunostaining for HPV shows no reactivity in any of the tumor cells (D).

**Figure 2:**
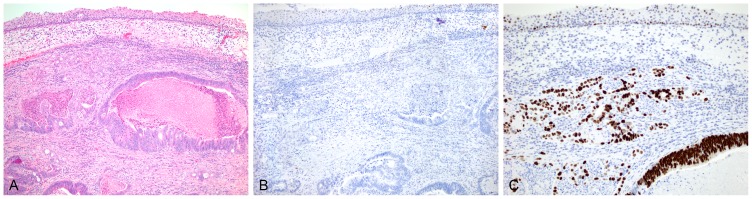
An H&E section of a separate case (pT2) shows adenocarcinoma underlying residual urothelium (A). This case shows no reactivity to p16 within the tumor or the normal urothelium with some faint nonspecific background staining seen (B). Very strong nuclear reactivity to p53 is seen in essentially all tumor cells. Weak-to-moderate reactivity is seen within the overlying urothelial cells (C).

**Figure 3:**
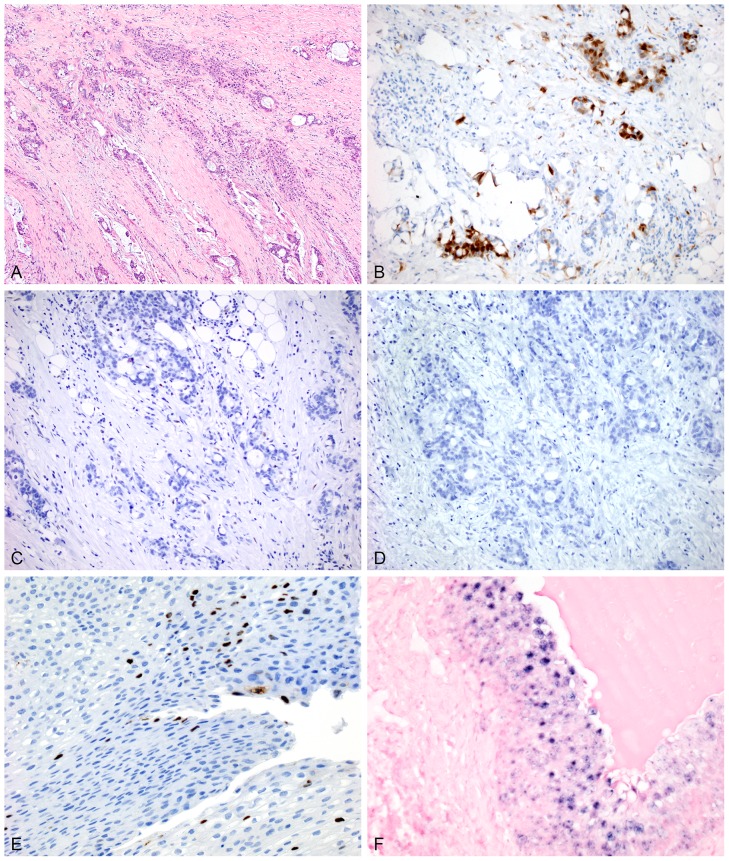
One additional case of primary adenocarcinoma of the urinary bladder (pT3) is highly infiltrative with some mucinous features on H&E (A). p16 shows strong nuclear and cytoplasmic reactivity in a majority of cells, but there is some variability in the staining (B). No reactivity is seen with either p53 (C) or HPV (D) immunostains in this case. Controls for HPV immunohistochemistry (E) and in situ hybridization (F) are demonstrated here.

Strong nuclear p16 expression was seen in 67% (24 of 36) of the cases and p53 expression was present in 58% (21 of 36). Expression of both markers was observed in 33% (12 of 36) of the cases (**Figure 1**). Expression of p16 or p53 alone was seen in 12 (33%) and 9 (25%), respectively (**Figures 2** **and** **3**). In 3 tumors (8%), labeling for both markers was negative. In addition, two cases were found to have strong cytoplasmic p16 staining, without nuclear staining, and were disregarded as “positive” in our results. In all 36 cases, HPV-ISH and HPV immunohistochemistry were completely nonreactive with working controls (**Figures 3D–F**). With regard to extent of staining, p53 staining was typically either very strong (3+) or nonreactive with 15 cases each showing these patterns. The remaining six cases demonstrated 2+ reactivity. Staining for p16 demonstrated far greater variability. In the 24 cases with p16 staining, only 1+ reactivity was seen in 11 of the cases. Tumors found to have 2+ and 3+ reactivity were seen in 5 and 8 cases, respectively (**Table 2**). Staining intensity was strong and relatively uniform in all cases where reactivity was reported. Though nearly all cases lacked uninvolved urothelium for comparison, one case did show nondiffuse staining for p53 in the urothelium of a weaker intensity than seen in the tumor; no staining was seen for p16 (**Figure 2B–C**).

**Table 2:** Tumor Staining Characteristics.

Antibody or ISH method used	p16	p53	HPV IHC*	HPV-ISH†
*Staining extent* ‡				
0	12	15	36	36
1+	11	0	0	0
2+	5	6	0	0
3+	8	15	0	0
*Any reactivity by tumor stage*				
pT1	7	10	0	0
pT2	6	5	0	0
pT3	8	5	0	0
pT4	3	1	0	0

*IHC = immunohistochemistry.

† ISH = in situ hybridization.

‡ Staining extent is defined within the “Methods” section of the text.

When analyzing for correlations between the markers, a slight inverse correlation between p16 and p53 expression patterns was seen, however, it lacked statistical significance (correlation coefficient = −0.1; p>0.5). An inverse correlation between p53 expression and tumor stage was seen (correlation coefficient = −0.2; p<0.01), while a slight positive correlation between p16 expression and stage was present (correlation coefficient = 0.16; p<0.01) (**Table 2**). It should, however, be made clear that these results are on the entire population and confounded by the large variability in staging characteristics between cystectomy and non-cystectomy specimens. Nodal disease was only detected in two of the cases, both showing p16 expression without p53 expression. The great majority of the cases in this series (partially due to the number of non-cystectomy cases) did not contain evaluable lymph nodes and prevented a more robust correlation with nodal disease. Long-term follow up on these cases was severely limited due to the multi-institutional nature of the study and broad timeline required to amass this amount of cases in such a rare entity.

## Discussion

In primary urinary bladder adenocarcinomas, expression of p16 and/or p53 is frequently present. In the sporadic cases studied here, 95% showed expression of at least one of the markers. These findings corroborate the high degree of p53 expression seen in the prior study by Kapur *et al.* and confirm that tumor suppressor proteins are commonly expressed in non-bilharziasis bladder adenocarcinoma [14]. To our knowledge it is also the first study to demonstrate a high degree of p16 expression in primary adenocarcinoma of the urinary bladder. The expression of these two cell cycle regulators in these tumors is similar to the frequency reported for other types of urothelial carcinoma and likely implies that mutations in genes regulating these proteins are involved in the development or progression of both conventional urothelial and adenocarcinoma of bladder tumors [12], [13].

Alterations in p53 have been studied in a number of human malignancies. The protein is a key regulator of the cell cycle. Alterations in p53 function leads to inhibition of apoptosis and increased cellular proliferation [20]. Alteration in p53 has been well studied in urothelial carcinoma. Its overexpression correlates with higher tumor grade and stage, disease progression and decreased survival [12], [13], [21]. Its status may also predict response to therapy. Tumors with p53 alteration are associated with chemoresistance [22]. Furthermore, studies examining p53 in mouse models have shown that deficiencies in p53, in concert with Rb1 alteration, were necessary, but not sufficient, for initiation of urothelial tumorigenesis. Loss of either regulatory pathway, without loss of the other, failed to increase susceptibility to urothelial malignant transformation in the mouse [23].

Rb1, the protein product of the *RB* (retinoblastoma) gene, is the major regulatory target for p16 within the cell cycle and is inactivated by HPV [24], [25]. In the hypophosphorylated state, Rb1 allows a cell to pass the G1/S checkpoint and proceed to DNA replication for cell division [26]. As both Rb1 and p16 are tumor suppressor genes, alterations in one or both proteins disrupt the cellular mechanisms available to halt tumor proliferation. A functional loss of Rb1 has been described to cause a positive feedback loop with p16 leading to accumulation of the protein (hence, immunohistochemical overexpression) without ability to halt the cell cycle; essentially negating its “tumor suppressor” ability [27]. Alterations of Rb1 and subsequent overexpression of p16 have been described in a number of non-HPV driven tumors, as well [27]. Overexpression of Rb1 and p16 are associated with a poor prognosis in the setting of nonsmall cell lung carcinoma [28]. Therefore, alterations in Rb1 or p16 may lead to overexpression via immunohistochemistry, with or without HPV infection.

Just over 75% of our cases showed increased expression of p16. This frequency is higher than the reported rate of p16 expression in urothelial carcinoma in studies from Tzai *et al.* (20% p16 positive) or Shariat et al. (54% p16 altered) [12], [13]. Neither study found any statistical significance relating p16 expression and disease status [12], [13]. A recent investigation of p16 in urothelial carcinoma in situ also showed a high rate of p16 expression without HPV association [29]. In this study, however, Steinestel *et al.* were able to demonstrate a link between p16 overexpression and increased cell signaling for epithelial-mesenchymal transition. There was no FISH evidence of direct amplification of the *p16* gene, suggesting that the p16 staining reflected malfunction of the Rb1 pathway [29].

The high rate of p16 expression observed in these tumors raises the possibility that unsubstantiated conclusions about the relationship between HPV and primary bladder adenocarcinoma may be inferred. The use of p16 staining in squamous neoplasia of the uterine cervix or head and neck has gained widespread clinical use in recent years. Its utility as a surrogate for HPV etiology is supported by a wealth of literature [17]–[19], [30]–[34]. Thus, p16 might easily be misinterpreted by clinicians or pathologists as a *de facto* HPV marker. To fully evaluate this potential, we also employed HPV immunohistochemistry and HPV-ISH in all tumors. No HPV virus protein or gene signature was detected in any case. This strongly suggests that the virus is not involved in the tumorigenesis of primary adenocarcinoma of the urinary bladder. HPV infection has been reported in the setting of bilharziasis-associated urothelial carcinoma. In one study HPV genetic material was present in 7 of 10 cases of schistosomal cystitis with dysplasia, but in none of 5 cases with nonspecific cystitis. However, there was no correlation between high or low risk HPV type and urothelial neoplasia, suggesting that any HPV involvement in schistosome-associated bladder cancer must involve different molecular mechanisms than in uterine cervix or upper aerodigestive mucosa tumorigenesis [35]. Other studies examining a multitude of non-bilharziasis associated urothelial neoplasms have not revealed the presence of HPV infection [11], [36], [37].

In summary, p16 and p53 are expressed in a high proportion of urinary bladder primary adenocarcinomas, often with coexpression of both tumor suppressor proteins. Considering these findings, it is likely that alterations in these cell cycle proteins contribute to the biological mechanisms driving these tumors. In addition, HPV infection was not identified in any case. Therefore, p16 expression should not be interpreted as a surrogate for HPV infection in these tumors. These findings help to advance the relative paucity of data available regarding the molecular and cellular events leading to the initiation and progression of bladder primary adenocarcinoma. Further investigation of cell cycle proteins and elucidation of molecular alterations of this tumor are necessary to further characterize its development and hopefully leads to better prognostication and treatment.
